# Panoramic ultrasound vs. MRI for the assessment of hamstrings cross-sectional area and volume in a large athletic cohort

**DOI:** 10.1038/s41598-020-71123-6

**Published:** 2020-08-24

**Authors:** Martino V. Franchi, Daniel P. Fitze, Jonas Hanimann, Fabio Sarto, Jörg Spörri

**Affiliations:** 1 Department of Biomedical Sciences, Institute of Physiology, University of Padua, Padua, Italy; 2 Sports Medical Research Group, Department of Orthopaedics, Balgrist University Hospital, University of Zurich, Zurich, Switzerland; 3 University Centre for Prevention and Sports Medicine, Balgrist University Hospital, University of Zurich, Zurich, Switzerland

**Keywords:** Musculoskeletal system, Medical imaging

## Abstract

We investigated the validity of panoramic ultrasound (US) compared to magnetic resonance imaging (MRI) for the assessment of hamstrings cross-sectional area (CSA) and volume. Hamstrings CSA were acquired with US (by an expert operator) at four different sites of femur length (FL) in 85 youth competitive alpine skiers (14.8 ± 0.5 years), and successively compared to corresponding scans obtained by MRI, analyzed by a trained vs. a novice rater. The agreement between techniques was assessed by Bland–Altman analyses. Statistical analysis was carried out using Pearson’s product moment correlation coefficient (*r*). US-derived CSA showed a very good agreement compared to MRI-based ones. The best sites were 40% FL (0 = mid patellar point) for biceps femoris long head (r = 0.9), 50% for semitendinosus (r = 0.9), and 30% for semimembranosus (r = 0.86) and biceps femoris short head (BFsh, r = 0.8). US-based vs. MRI-based hamstrings volume showed an r of 0.96. Poorer r values were observed for the novice compared to the trained rater, with the biggest difference observed for BFsh at 50% (r = 0.001 vs. r = 0.50, respectively) and semimembranosus at 60% (r = 0.23 vs. r = 0.42, respectively). Panoramic US provides valid CSA values and volume estimations compared to MRI. To ensure optimal US-vs.-MRI agreement, raters should preferably possess previous experience in imaging-based analyses.

## Introduction

The assessment of skeletal muscle size is central within many athletic performance and clinical scenarios. Monitoring changes in muscle size allows to gain more specific insights into the actual status of athletes within their long-term athletic development process or their return-to-sport journeys, when recovering from injuries^1^. The most commonly used measures of muscle size are muscle cross-sectional area (CSA) and muscle volume. Muscle CSA has been widely used in research and sport medicine contexts being strongly related to joint torque production in both quadriceps and hamstrings muscle groups^2,3^. Furthermore, muscle CSA assessment has been recently shown to be useful in hospital settings for predicting survival and risk of treatment failure^4^ as well as for improving the prognosis process when evaluated overtime^5^. Similarly, muscle volume is regarded as one of the best predictors of joint torque in humans for both upper and lower limbs of healthy males and sport athletes^6,7^.

The quantification of muscle CSA and volume is usually obtained by imaging techniques, such as computer tomography (CT) and magnetic resonance imaging (MRI). The latter is recognized as the gold standard for clinical and research imaging, providing accurate estimations of muscle size and involving minimal radiation exposure compared to CT scans^8^. However, MRI is not cheap and not always accessible as other imaging techniques^9^, especially in athletic-related settings, when many measures should be acquired over time (and preferably independently of fixed imaging facilities) in order to meticulously monitor the athletic development process or the successfulness of return to sport programs.

In such context, ultrasound (US) could represent a cheaper, yet reliable alternative for the quantification of muscle size. Specifically, after the introduction of panoramic ultrasound imaging (i.e. the Extended Field of View—EFOV technique)^10^, sonography can be adopted to measure larger anatomical structures, such as full muscle CSAs when scans are acquired in the transversal plane^11^, as it has been previously described in two pioneering studies investigating the quadriceps muscle group^12,13^.

In the present investigation we have focused on the use of panoramic US (i.e. compared to MRI-based imaging) for the evaluation of hamstrings muscles CSA and volume in a large athletic cohort. Previously, individual hamstrings muscle CSA and volume have been assessed across maturation and athletic development^14^, while recovering from anterior cruciate ligament reconstruction (ACLR) or hamstring strains^15–17^, and before return-to-sport clearance^1^. Moreover, with respect to both ACLR and hamstring strains, substantial reductions of muscle volume in biceps femoris long head (BFlh) or semitendinosus (ST) have been observed, and monitoring muscle size becomes even more imperative in situations where a re-injury may occur^18–20^. In the specific context of competitive alpine skiers, hamstrings muscles play an especially important role, as they may act as an ACL-synergist by producing a posteriorly directed shear force to the tibia (i.e., by eccentrically resisting the boot-induced anterior drawer of the tibia relative to the femur that is known to be typical for skiing-related ACL injury mechanisms)^21–24^. In all of these examples, the US-based evaluation of hamstrings CSA and volume may serve as a meaningful monitoring tool.

Therefore, the main objectives of our study were to investigate the validity of panoramic US compared to MRI for the assessment of (1) individual hamstring muscles CSA and (2) hamstring muscles volume in a large athletic cohort of 85 youth (13–15 years old) competitive alpine skiers. Additional objectives in subgroups of the entire athletic cohort were: (3) to assess the intra-session test–retest reliability of the US-derived CSA acquisition and assessment; (4) to compare the US-derived CSA post-acquisition evaluation (contour tracing) of a trained vs. a complete novice rater (inter-rater reliability); and (5) to explore the differences in US-derived CSA contour tracing of the trained rater before and after the MRI analyses being carried out (effect of image-analysis training).

## Methods

Throughout the manuscript, all values are presented as means ± SD.

We recruited 85 youth competitive alpine ski racers (36 females, 49 males—14.8 ± 0.5 years old, height 166 ± 7.6 cm, body weight 56.6 ± 9 kg) that were part of a certificated regional performance center (RLZ/CRP) of Swiss-Ski, i.e. representing the best level skiers of that age-group in Switzerland. Measurements were acquired during the preseason period (October–December). The protocol was approved by the institutional review board at Balgrist University Hospital and the local ethics committee (KEK-ZH-NR: 2017-01395). All subjects gave written informed consent in accordance with the Declaration of Helsinki.

### Ultrasound imaging

All ultrasound images were acquired by an expert operator (MVF) with the same ultrasound device throughout the whole study (Aixplorer Ultimate, SuperSonic Imagine, Aix-en-Provence, France) using a linear 50 mm transducer (SuperLinear SL18-5, SuperSonic Imagine, Aix-en-Provence, France). Participants were asked to lie prone on a massage bed and were instructed to rest with extended knee joints, and to relax completely during image acquisition with their feet placed just outside the bed frame in order to avoid any lower limb imbalances. Five minutes of rest in this position were provided for body fluid shift stabilization.

For the panoramic ultrasound scans, the region of interest (ROI) was determined and marked as follows. Firstly, a mark was drawn at 50% of the distance between the greater trochanter and the mid patella point. In second instance, other marks were drawn with a permanent ink pen at the 30%, 40% and 60% of the femur length (distal to proximal, thus 0% was representing the mid patellar point and 100% the great trochanter). The ROIs were identified laterally, from the borders of vastus lateralis muscle, and medially, until the borders of the gracilis muscle, similar to the guidelines suggested by Balius and colleagues^25^. Multiple ROIs were identified in a similar fashion to the study of Kositsky and colleagues^26^, the reason being that distinct muscles present their CSA peak value at distinct muscle sites. In addition, obtaining CSA values at multiple sites allowed us to reconstruct a bigger portion of muscle volume of the hamstrings muscle group.

At each ROI, the transducer was placed on the lateral portion of the posterior thigh (just before the Biceps femoris long and short heads muscle borders) and then moved on the transversal plane in a lateral-to-medial fashion until the end of borders of the semimembranosus and the start of gracilis muscle were identified, then the panoramic acquisition was stopped. We have carefully ensured that the images were collected at the right angle in the transverse plane by using a plastic guide placed on the skin of the volunteer’s thigh, similar to the one used by Noorkoiv et colleagues^13^. The transducer was kept in contact with the guide throughout the whole acquisition of CSAs, thus we ensured that the right CSA path was followed while keeping the transducer perpendicular to the skin. The operator took meticulous care in keeping the pressure as constant as possible during the entire image acquisition. For all scans, transmission gel was used to improve the acoustic contact and to keep the pressure on the skin to a minimum. For the purpose of test–retest reliability assessment as outlined below, EFOV scans were acquired twice after completely removing the transducer from the skin and with 5 min rest period in between.

Ultrasound images were analyzed in randomized order by tracing the contours of each four of the hamstring muscles (BFlh, BFsh, ST, SM) at each ROIs using Image J, a public domain software for image analysis (https://imagej.nih.gov/ij/). Once CSAs measurements were obtained at the specific ROIs along the muscle length, by knowing the distance between the different ROIs (constant, as they were identified as percentages of the total femur length), muscle volume (VOL) was calculated for each muscle (for the portion between 30 and 60% of whole femur length) using the truncated cone formula^27,28^:

$$ {\text{VOL}} = 1/3*{\text{h}}*[{\text{CSA}}_{1} + {\text{CSA}}_{2} + \surd ({\text{CSA}}_{1} *{\text{CSA}}_{2} )] $$

where h is the distance between CSA_1_ and CSA_2_.

### Magnetic resonance imaging

**Figure 1:**
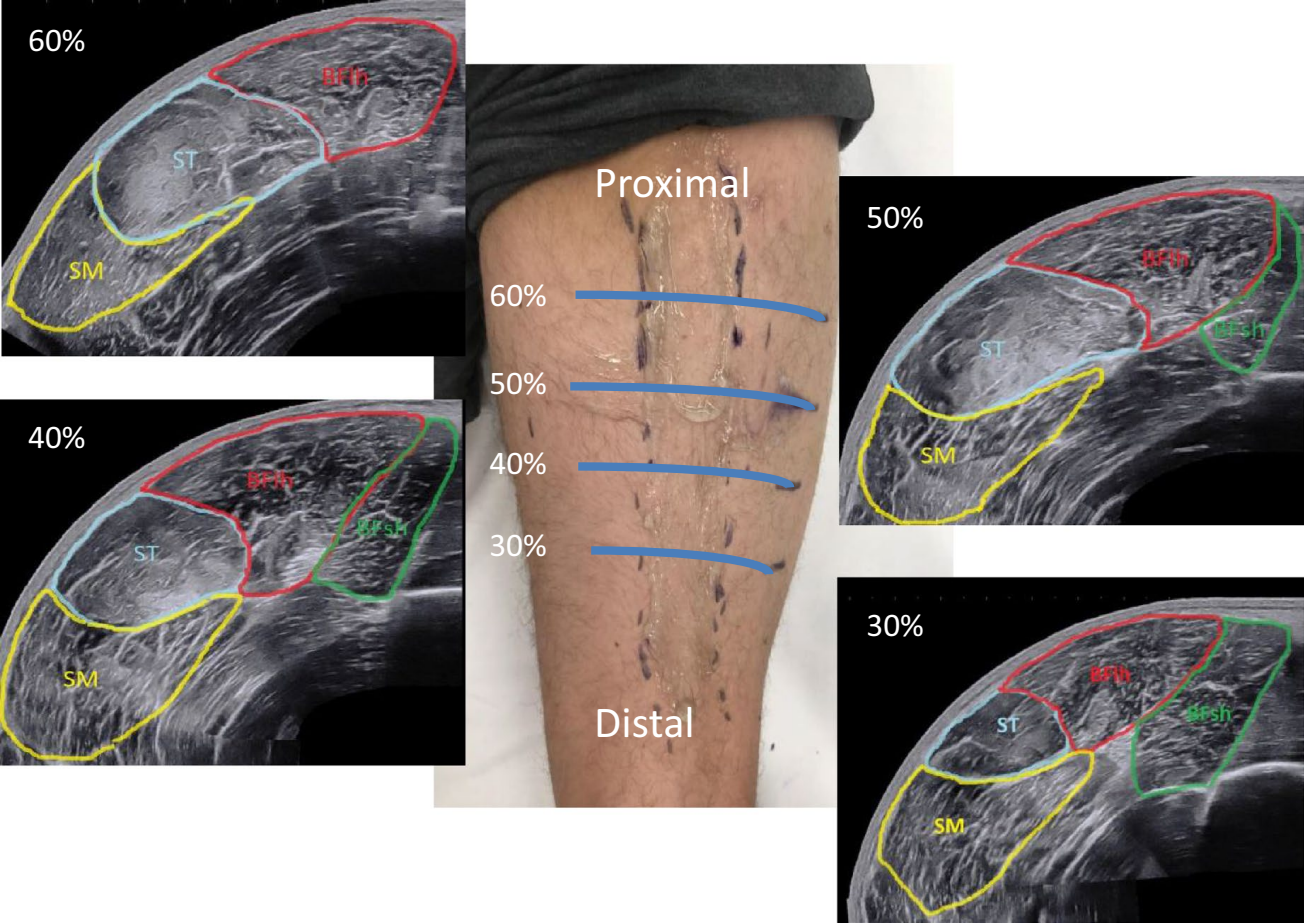
The four measurement sites (ROIs) at different portion of the femur length and the relative panoramic US scans of hamstring muscles. The picture was taken by MVF and DPF at Uniklinik Balgrist, Swiss Centre for Musculoskeletal Imaging, Zurich, Switzerland.

All MRI data were acquired on a 3T scanner (Magnetom Prisma, Siemens, Erlangen, Germany). Participants were positioned supine on the MR system's patient bed, fitted with ear protection, and instructed regarding the use of the alarm bell and scans, followed by the acquisition of localizer scans. After ca. 5 min that allowed for body fluid shift stabilization, up to three spatially overlapping T1w spoiled 3D gradient echo data sets of both thighs were acquired in the axial (transverse) orientation and combined into a single image series. Acquisition parameters: sequence: vibe, TR/TE: 3.23/1.23 ms, flip angle: 15°, field of view: 450 × 337.5 mm, encoded nominal voxel size: 1.3 × 1.3 × 3 mm, no inter-slice gap, readout-bandwidth 790 Hz/pixel (278 kHz), acquisition time per 3D volume: 1 min 23 s. In the corresponding images, contours of the semimembranosus (SM), the semitendinosus (ST), and the biceps femoris long (BFlh) and short heads (BFsh) muscles were digitized in randomized order using the Merlin Diagnostic Workcenter DICOM image analysis software (Phönix-PACS GmbH, Version 5.3.156494, https://www.phoenix-pacs.de/image-display/) (Fig. 1). As for the US technique, once CSAs measurements were obtained at the specific ROIs along the muscle length, muscle volume (VOL) was calculated using the truncated cone formula^27,28^ for each muscle (for the portion between 30 and 60% of whole femur length).

### Statistical analyses

Data analysis was performed using GraphPad Prism software (version 8.00, GraphPad Software, San Diego California USA).

#### Comparison of a panoramic US- vs. MRI-derived hamstrings CSA and volume assessment (objective 1 and 2)

US- and MRI-derived hamstrings CSA and hamstrings muscle volume data were reported as mean ± SD. Normality of distribution was checked by the Shapiro–Wilk’s test. The agreement between techniques was assessed by using Bland–Altman analyses^29,30^. The validity between MRI and US was tested by plotting the differences in CSA measurements by both techniques against their means, setting ± 1.96 SD as the limits of agreement^12^. If the differences were within ± 1.96 SD, then the two methods can be used with similar accuracy to measure muscle CSA. Correlations were tested by using the Pearson’s product moment correlation coefficient (*r*) and their 95% confidence intervals (Table 1). The level of significance was set at *p* < 0.05.

**Table 1:** Parameters of level of agreement derived from Bland Altman analyses for MRI and US measurements of hamstring muscles CSA at different portions of femur length.

Rater 1—MRI vs. US (MRI CSAs evaluated post US assessment, both in randomized order)					
Muscle (distal—0%, to proximal—100%)	MRI CSA (cm^2^)	Mean bias (cm^2^) [%]	ULOA (cm^2^) [%]	LLOA (cm^2^) [%]	Pearson’s *r *(95% CI)
**Semimembranosus**					
30% (n = 85)	10.07 ± 2.32	0.02 ± 1.2 [− 0.4%]	2.38 [24%]	− 2.37 [− 25%]	0.86 (0.79–0.90)
40% (n = 85)	8.09 ± 2.19	− 0.77 ± 1.38 [− 9%]	1.94 [23%]	− 3.5 [− 42%]	0.79 (0.69–0.86)
50% (n = 85)	4.51 ± 1.98	− 1.24 ± 1.37 [− 25%]	1.44 [49%]	− 3.94 [− 99%]	0.78 (0.68–0.85)
60% (n = 70)^a^	1.61 ± 1.15	− 0.99 ± 1.09 [− 53%]	1.15 [52%]	− 3.14 [− 159%]	0.64 (0.48–0.76)
**Semitendinosus**					
30% (n = 85)	4.17 ± 1.82	0.33 ± 0.96 [3%]	2.23 [74%]	− 1.55 [− 67%]	0.84 (0.77–0.89)
40% (n = 85)	7.33 ± 1.72	0.44 ± 1.04 [7%]	2.48 [38%]	− 1.6 [− 23%]	0.84 (0.77–0.89)
50% (n = 85)	8.64 ± 2	0.26 ± 0.92 [4%]	2.08 [27%]	− 1.55 [− 19%]	0.90 (0.86–0.93)
60% (n = 85)	8.5 ± 2.01	− 0.34 ± 0.99 [− 4%]	1.6 [18%]	− 2.29 [− 26%]	0.87 (0.82–0.92)
**Biceps femoris long head**					
30% (n = 85)	8.12 ± 2.26	1.19 ± 1.19 [16%]	3.54 [48%]	− 1.15 [− 16%]	0.85 (0.78–0.90)
40% (n = 85)	10.36 ± 2.19	0.77 ± 0.91 [8%]	2.56 [26%]	− 1.02 [− 10%]	0.90 (0.86–0.94)
50% (n = 85)	8.93 ± 1.84	− 0.36 ± 0.98 [− 4%]	1.55 [19%]	− 2.28 [− 27%]	0.83 (0.75–0.89)
60% (n = 84)^a^	5.96 ± 1.73	− 0.91 ± 1.16 [− 15%]	1.35 [22%]	− 3.18 [− 54%]	0.75 (0.64–0.83)
**Biceps femoris short head**					
30% (n = 85)	5.71 ± 1.38	− 0.08 ± 0.91 [− 1%]	1.7 [34%]	− 1.88 [− 36%]	0.80 (0.71–0.86)
40% (n = 85)	3.71 ± 1.03	− 0.23 ± 0.83 [− 6%]	1.4 [37%]	− 1.88 [− 49%]	0.69 (0.56–0.79)
50% (n = 82)^a^	1.85 ± 0.74	− 0.63 ± 0.83 [− 29%]	1 [40%]	− 2.26 [− 99%]	0.37 (0.17–0.55)
60% (n = 0)^a^		–	–	–	–

Pearson’s r correlation coefficients are reported too.

*ULOA *upper limit of agreement, *LLOA *lower limit of agreement.

^a^Lower number of subjects due to not visible muscle

#### Test–retest reliability US-derived CSA assessment (objective 3)

The intra-session test–retest reliability of hamstrings muscles CSA measured by US was analyzed based on the data of 6 subjects randomly chosen from the entire athletic cohort. The test–retest reliability assessment for EFOV ultrasound technique was performed at one specific ROI (i.e., the 50% of femur length) by calculating the intraclass correlation coefficient (ICC) and their 95% confidence intervals based on a mean measurement absolute-agreement, two-way mixed model effect (ICC_3,k_), for all muscle architecture parameters, as previously performed by Kositsky and colleagues^26^. Test–retest reliability was classified as good (0.75–0.90) and excellent (> 0.90), following the classification proposed by Ko and Li^31^.

#### Inter-rater reliability of the US-derived CSA assessment: trained rater vs. complete novel rater (objective 4)

In a subgroup of 45 randomly chosen youth athletes we compared the differences in US-derived CSA contour tracing between two operators (trained—rater 1 vs. completely novel—rater 2) for the same ultrasound images for all the hamstrings muscles at all the ROIs. We decided to select n = 45 youth athletes as the half of the total number (n = 85) of subjects investigated rounded up to n = 45. Image analysis was blinded and performed in a carried out in a randomized order. The agreement between techniques was assessed by using Bland–Altman analyses for both raters. Correlations were tested by using the Pearson’s product moment correlation coefficients (*r*) and their 95% confidence intervals. Further, Pearson’s product moment correlation coefficient (*r*) were transformed in Fisher’s z-scores and the difference for all z-scores at all muscle sites were tested by paired t-test between the two raters (Table 4). The level of significance was set at *p* < 0.05.

#### Differences in US-derived CSA assessment of the trained rater before and after the MRI analyses being carried out (objective 5)

Also, in the same subgroup of 45 randomly chosen youth athletes we compared the differences in US-derived CSA contour tracing of the same rater before and after carrying out the MRI analyses. For clarity, the rater first analyzed US scans, then MRI scans, and lastly US images again. Image analysis was blinded and performed in a randomized order for all the three distinct batches of data analysis. The agreement between techniques was assessed by using Bland–Altman analyses for both rater 1 assessments (pre vs. post MRI analyses). Correlations were tested by using the Pearson’s product moment correlation coefficient (*r*) and their 95% confidence intervals. Further, Pearson’s product moment correlation coefficients (*r*) were transformed in Fisher’s z-scores and the difference for all z-scores at all muscle sites were tested by paired t-test between the two raters (Table 4). The level of significance was set at *p* < 0.05.

## Results

### Comparison of a panoramic US- vs. MRI-derived hamstrings CSA and volume assessment

The average CSA values for each muscle at each specific ROI (evaluated by a trained rater—rater 1) are presented for MRI and US in Table 1 together with the results of the Bland Altman (mean bias and limits of agreements) and Pearson’s correlation analyses. In general, a very good agreement was observed between the two techniques for each hamstrings muscle. Pearson’s correlation coefficient ranged from 0.64–0.86 for SM (p < 0.0001 for all ROIs), from 0.84–090 for ST (p < 0.0001 for all ROIs), from 0.75–0.90 for BFlh (p < 0.0001 for all ROIs), and from 0.37–080 for BFsh (p < 0.0001 for 30% and 40% ROIs, p < 0.01 for 50% ROI).

ST and BFlh were the muscles that presented the highest r values at the ROIs of 50% and 40% of femur length, respectively (r = 0.90 for both muscles at those specific ROIs). The muscles that presented the lowest r values between US and MRI were SM and BFsh at the ROIs of 60% and 50% of the femur length, respectively (r = 0.64 and r = 0.37, mean % bias = − 53% and − 29%, respectively). Over the specific ROI in which the scans were acquired (between 30 and 60% of femur length, mid patellar point regarded as 0%), maximal CSA measured from MRI was 8.09 ± 2.19 for SM located at the 40%, 8.64 ± 2 for ST located at the 50%, 10.36 ± 2.19 for BFlh located at the 50%, and 5.71 ± 1.38 for BFsh located at the 30%.

**Figure 2:**
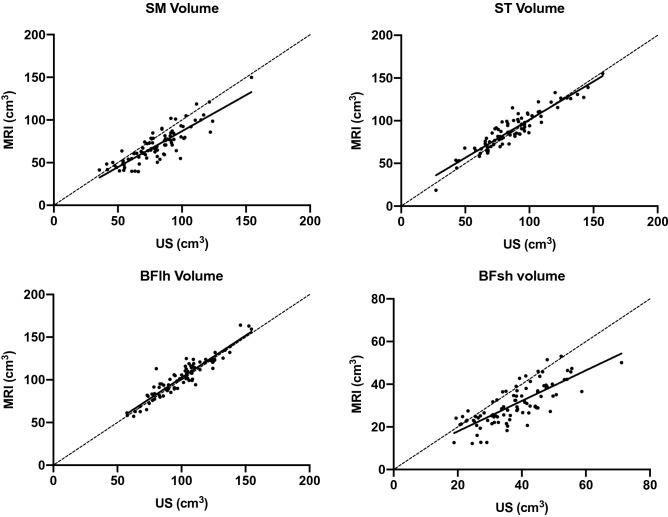
Correlations between MRI and US measurements for the assessment of muscle volume of each hamstring muscles. The volume is representative of the muscle portions scanned, thus between 30 and 60% of femur length.

**Figure 3:**
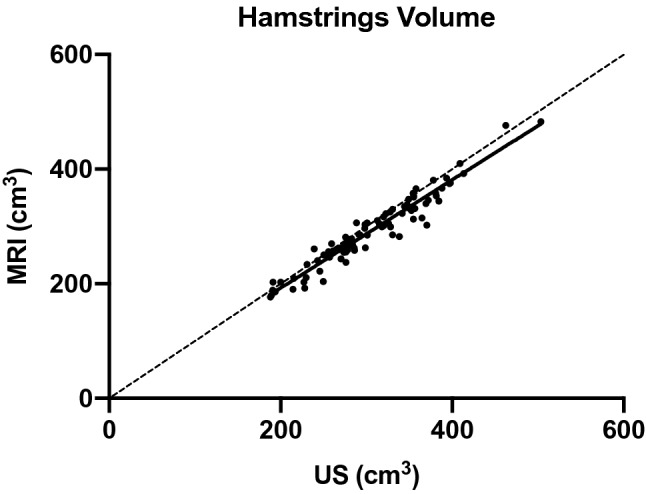
Correlation between MRI and US measurements for the assessment of muscle volume of hamstring muscles pooled together. The volume is representative of the muscle portions scanned, thus between 30 and 60% of femur length.

The comparisons between volume calculations (for the muscle portions scanned between 30 and 60% of femur length) carried out by rater 1 from all the US vs. MRI-derived CSA analyses are presented for each muscle (Fig. 2) and for all the hamstrings pooled together (Fig. 3). Pearson’s r values were r = 0.86 for SM, r = 0.93 for ST, r = 0.93 for BFlh, and r = 0.78 for BFsh. For all the hamstrings pooled together, the r value for hamstrings volume was 0.96 between US and MRI analyses.

### Test–retest reliability US-derived CSA assessment

The intra-session reliability data (ICCs_3,k_, Pearson’s correlations and SEMs) for each muscle CSA acquired by EFOV US technique at the 50% of femur length are presented in Table 2. SM, ST and BFlh muscles showed a very high relative reliability (ICC_3,k_ 95% CI 0.74–0.99, 0.93–0.99, and 0.87–0.99, respectively). A high repeatability was also observed for BFSh CSA measurement (ICC_3,k_ 95% CI 0.36–0.98).

**Table 2:** Intra-session repeatability for CSA values for each muscle at the 50% of femur length region of interest, measured by EFOV technique within the same session (n = 6, 15 min between each measure).

Muscle	Measure1 (cm^2^)	Measure2 (cm^2^)	ICC_3,k_ (95% CI)	SEM (cm^2^)
SM	6.11 ± 1.57	5.94 ± 1.46	0.96 (0.74–0.99)	0.41
ST	9.53 ± 2.88	9.77 ± 2.95	0.99 (0.93–0.99)	0.19
BFlh	9.58 ± 2.25	9.64 ± 1.94	0.97 (0.87–0.99)	0.57
BFsh	2.21 ± 0.61	1.97 ± 0.6	0.85 (0.36–0.98)	0.17

*ICC *intraclass correlation coefficient (with 95% confidence intervals), *SEM *standard error of the mean.

### Inter-rater reliability of the US-derived CSA assessment

The US-derived CSA values and the Pearson’s correlation coefficients for rater 1 (trained) and rater 2 (completely novel) (relative US vs. MRI comparison) are shown in Table 3 for a sub-cohort of 45 athletes. Generally, worse Pearson’s r values were observed for rater 2 compared to rater 1, the biggest difference being represented by BFsh values at 30% of femur length (r = 0.001 vs. r = 0.50, respectively) and SM at 60% (r = 0.23 vs. r = 0.42, respectively). The Fisher’s z-score for the same analyses are shown in Table 4; rater 1 shows a higher overall z-score compared to rater 2 (p < 0.001).

**Table 3:** (Left portion) Comparison of the digitation of muscle contours from US between a trained and a novice rater. Presented are the absolute levels of agreement derived from Bland Altman analyses (with lower and upper limits of agreement) and Pearson’s correlation coefficients (r) (with 95% confidence intervals) of MRI and US measurements for hamstring muscles CSA at different portions of femur length. (Right portion) Absolute level of agreement and Pearson’s correlation coefficients (r) (with 95% confidence intervals) between MRI and US measurements for the trained rater after the analysis of MRI scans.

Rater 1 (trained) vs. Rater 2 (novice) (US) (MRI CSAs assessed in randomized order by rater 1 post US assessment n1)					Rater 1 (trained) assessment n2 (US) (US CSAs re-assessed in randomized order post MRI assessment)	
Muscle (distal—0%, to proximal—100%)	Rater 1 Mean bias (LLOA –ULOA) (cm^2^)	Rater 1 Pearson’s r (95% CI) (US vs. MRI)	Rater 2 Mean bias (LLOA –ULOA) (cm^2^)	Rater 2 Pearson’s r (95% CI) (US vs. MRI)	Rater 1 US CSA—n2 Mean bias (LLOA –ULOA) (cm^2^)	Rater 1 Pearson’s r (95% CI) (US-2 vs. MRI)
**Semimembranosus**						
30% (n = 45)	− 0.17 (− 2.54 to 2.20)	0.90 (0.83 to 0.94)	− 0.59 (− 4.36 to 3.18)	0.76 (0.59 to 0.86)	0.09 (− 1.54 to 1.73)	0.95 (0.92 to 0.97)
40% (n = 45)	− 0.53 (− 3.02 to 1.94)	0.85 (0.75 to 0.92)	− 0.74 (− 3.51 to 2.01)	0.85 (0.73 to 0.91)	− 0.53 (− 1.80 to 1.23)	0.94 (0.90 to 0.97)
50% (n = 45)	− 1.01 (− 3.70 to 1.78)	0.81 (0.69 to 0.89)	− 0.96 (− 4.97 to 3.03)	0.69 (0.49 to 0.81)	− 0.59 (− 2.66 to 1.58)	0.86 (0.75 to 0.92)
60% (n = 40)^a^	− 1.04 (− 3.51 to 1.41)	0.42 (0.13 to 0.65)	− 1.16 (− 4.36 to 2.02)	0.23 (− 0.01 to 0.50)	− 1.12 (− 3.71 to 1.46)	0.41 (0.11 to 0.64)
**Semitendinosus**						
30% (n = 45)	0.19 (− 1.73 to 2.12)	0.84 (0.73 to 0.91)	0.68 (− 1.37 to 2.75)	0.83 (0.70 to 0.90)	0.38 (− 1.06 to 1.83)	0.91 (0.85 to 0.95)
40% (n = 45)	0.39 (− 1.90 to 2.69)	0.84 (0.72 to 0.91)	0.61 (− 1.52 to 2.74)	0.87 (0.78 to 0.93)	0.51 (− 1.25 to 2.29)	0.90 (0.83 to 0.94)
50% (n = 45)	0.24 (− 1.54 to 2.06)	0.92 (0.87 to 0.95)	0.55 (− 1.87 to 2.98)	0.88 (0.79 to 0.93)	0.29 (− 1.01 to 1.58)	0.95 (0.92 to 0.97)
60% (n = 45)	− 0.19 (− 2.07 to 1.67)	0.90 (0.83 to 0.94)	− 0.22 (− 3.06 to 2.61)	0.77 (0.62 to 0.87)	− 0.23 (− 1.84 to 1.36)	0.93 (0.87 to 0.96)
**Biceps femoris long head**						
30% (n = 45)	1.21 (− 0.73 to 3.16)	0.89 (0.81 to 0.94)	0.84 (− 1.92 to 3.60)	0.82 (0.68 to 0.89)	1.09 (− 0.94 to 3.14)	0.88 (0.80 to 0.93)
40% (n = 45)	0.75 (− 1.42 to 2.93)	0.89 (0.81 to 0.94)	0.24 (− 2.12 to 2.60)	0.87 (0.77 to 0.92)	0.89 (− 0.87 to 2.66)	0.92 (0.87 to 0.96)
50% (n = 45)	− 0.22 (− 2.19 to 1.74)	0.88 (0.78 to 0.93)	− 1.11 (− 3.54 to 1.31)	0.84 (0.72 to 0.91)	− 0.07 (− 1.50 to 1.35)	0.93 (0.88 to 0.96)
60% (n = 45)	− 0.69 (− 3.19 to 1.79)	0.76 (0.60 to 0.86)	− 1.77 (− 4.68 to 1.13)	0.72 (0.53 to 0.83)	− 0.79 (− 2.97 to 1.38)	0.82 (0.70 to 0.90)
**Biceps femoris short head**						
30% (n = 45)	0.02 (− 1.89 to 1.93)	0.79 (0.65 to 0.88)	− 0.15 (− 2.32 to 2.01)	0.78 (0.63 to 0.87)	− 0.27 (− 1.42 to 0.86)	0.91 (0.85 to 0.95)
40% (n = 45)	− 0.08 (− 1.51 to 1.33)	0.74 (0.57 to 0.84)	− 0.04 (− 1.87 to 1.78)	0.64 (0.42 to 0.78)	− 0.02 (− 1.44 to 1.39)	0.78 (0.64 to 0.87)
50% (n = 36)^a^	− 0.65 (− 1.98 to 0.67)	0.50 (0.21 to 0.71)	− 0.68 (− 3.1 to 1.73)	− 0.001 (− 0.03 to 0.32)	− 0.25 (− 1.28 to 0.77)	0.82 (0.67 to 0.90)
60% (n = 0)^a^		–	–	–	–	–

^a^Lower number of subjects due to not visible muscle.

**Table 4:** Fisher’s z-scores (obtained from the transformation of the Pearson’s r values presented in Table 3) for the trained rater (rater 1), the novice rater (rater 2), and for the trained rater after MRI assessment.

Fisher’s z-scores Rater 1, rater 2, rater 1 assessment n2 (post MRI assessment)			
Muscle (distal—0%, to proximal—100%)	Rater 1 (trained)	Rater 2 (novice)	Rater 1 assessment n2
**Semimembranosus**			
30% (n = 85)	1.47	0.97	1.83
40% (n = 85)	1.25	1.22	1.73
50% (n = 85)	1.12	0.84	1.29
60% (n = 70)^a^	0.44	0.23	0.43
Mean ± SD	1.07 ± 0.44	0.81 ± 0.41	1.32 ± 0.63
**Semitendinosus**			
30% (n = 85)	1.22	1.18	1.52
40% (n = 85)	1.22	1.33	1.47
50% (n = 85)	1.58	1.37	1.83
60% (n = 85)	1.47	1.02	1.66
Mean ± SD	1.37 ± 0.18	1.22 ± 0.16	1.62 ± 0.16
**Biceps femoris long head**			
30% (n = 85)	1.42	1.12	1.37
40% (n = 85)	1.42	1.33	1.59
50% (n = 85)	1.37	1.22	1.65
60% (n = 84)^a^	0.99	0.88	1.15
Mean ± SD	1.30 ± 0.20	1.14 ± 0.19	1.44 ± 0.22
**Biceps femoris short head**			
30% (n = 85)	1.07	1.04	1.52
40% (n = 85)	0.95	0.75	1.04
50% (n = 82)^a^	0.54	0.01	1.15
Mean ± SD	0.85 ± 0.27	0.60 ± 0.53	1.24 ± 0.25
All muscles Mean ± SD	1.17 ± 0.33***	0.97 ± 0.39	1.42 ± 0.36***^,###^

*** p < 0.001 vs. rater 2, ^###^p < 0.001 for rater 1 n2 (post-MRI) vs. rater 1.

^a^Lower number of subjects due to not visible muscle.

### Differences in US-derived CSA assessment of a trained rater before and after the MRI analyses being carried out

The mean biases (with lower and upper limits of agreement) and the Pearson’s correlation coefficients for the trained rater's second US-derived CSA assessment and MRI (post MRI analyses) are also shown in Table 3, being carried out for each muscles and ROIs. Generally, after MRI evaluation better Pearson’s r values were observed, the biggest improvement being observed on BFsh at 30% and 50% ROIs (r = 0.79 vs. r = 0.91, and r = 0.50 vs. r = 0.82, respectively). The Fisher’s z-score for the same analyses are shown in Table 4; assessment number 2 (post MRI analyses) shows a higher overall z-score compared to assessment 1 (p < 0.001).

## Discussion

The main findings of the present study pointed out that US is valid and reliable for the assessment of individual hamstrings CSA and volume when compared to the gold-standard MRI. The Bland Altman analyses showed that the trained rater presented a better agreement (i.e., lower biases and narrower limits of agreements) between MRI and US-derived CSA values compared to a completely novice rater; further, it appears that is preferable to have previous experience in MRI-derived CSA evaluation.

### Methodological considerations: a valid and reliable assessment of US-derived hamstring CSA and volume assessment appears feasible

In first instance, our results revealed a good agreement between a panoramic US- vs. MRI-derived hamstrings CSA and volume assessments. Thus, compared to the golden reference standard MRI, a US-based approach can be considered being sufficiently valid. Moreover, the CSA agreement was better for specific muscles (ST and BFlh) at specific sites (50% and 40% of femur length, respectively). These data strongly support the previously published observations of Kositsky and colleagues^26^. In a very similar fashion, they showed that the highest agreement between the two imaging techniques was found at ROIs where CSA were the largest: in fact, in Kositsky et al. study the poorest agreement was found for BFsh muscle, especially closer to the 50% ROI (r = − 0.09), which was similar in the present investigation (r = 0.37). As Kositsky and colleagues explained in the discussion section of their study^26^, MRI shows low minimal detectable change (< ~ 7%) when assessing small muscles^32^. Therefore, errors in MRI-based CSA evaluation are unlikely to justify the large limits of agreements observed both in their and our studies. Nevertheless, the large relative differences in muscle CSA would be only found at sites where the CSA would be small, and this would represent less of a problem at ROIs of large CSA: in fact, better agreement was found for each muscle in the present study at large CSA sites (e.g. SM and BFsh at 30% of the femur length). In contrast with the previous work of Kositsky and colleagues^26^, we often observed a higher bias towards larger US values compared to MRI ones, especially for muscles that were more difficult to identify with US, usually for the presence of small image artifacts at specific ROIs, such as SM (at 50% and 60% and BFsh at 50%), which likely prevented the accurate identification and digitization of the CSA path. Therefore, we agree with Kositsky et al. that “interchanging and direct comparison of US and MRI measures from literature is inadvisable”.

With respect to reliability, the intra-session test–retest reliability US-derived CSA assessment was shown to be good to excellent (ICC ranging from 0.85 to 0.99) and, thus, confirmed previously reported ICCs for all hamstring muscles (0.88–0.99)^26^ and for ST, SM, and BFlh (0.92–0.98)^33^. Furthermore, in a sub-cohort of 45 athletes, the US vs. MRI agreement was observed to be higher for the trained vs. the novice rater, but, remarkably, the agreement also improved for the trained rater after carrying out the MRI analyses. Noteworthy, from the Bland Altman analyses we can notice that the difference of the mean biases between rater 1 and 2 was not observed to be as large as the ranges of the limits of agreement, suggesting that rater 2 was more prone to produce inconsistent US CSA traces when compared to MRI. This is supported by the significant difference found in the Fisher’s z-score for all the analyzed CSAs between rater 1 and 2 (Table 4). This methodological aspect acquires great practical impact, as novel operators should refrain from carrying out studies or clinical assessments if not properly trained. Moreover, it appears that previous experience with MRI-based analyses could represent an advantage even for trained ultrasonography operators.

### Sports practical and clinical importance: adding a seat to the table for panoramic US-derived hamstring CSA and volume assessments

The assessment of individual hamstring muscle CSA by MRI has been used in clinical and sportive settings in order to monitor either the successfulness of post-surgery treatments (e.g., after anterior cruciate ligament reconstruction involving a hamstring muscle graft^15,19,20,34^, or to identify potential risks of injury^17^. In such contexts, a US-derived hamstring CSA and volume assessment, possibly carried out with portable devices (and thus independent of imaging facilities), may open new athlete monitoring perspectives and possibilities. Specifically, for the cohort of the present study, physical aspects of the athlete have been suggested to be among the top 5 key injury risk factors in alpine ski racing^35^. During the “landing back weighted” ACL-rupture mechanisms, internal developed forces from the quadriceps muscles may strain the ACL in the distal range of motion close to full knee extension^36,37^, while the hamstring muscles act as an ACL synergist by producing a posteriorly directed shear moment on the tibia^38,39^. Thus, accurate values of muscle CSA can be very useful, being muscle strength previously related to changes in muscle size^2,3^. In fact, Konishi and Fukubayashi showed that the muscle torque per unit volume of patients with ACLR at 12 months in both injured, and uninjured sides was significantly lower than those of controls^40^. Furthermore, a marked reduction of ST volume (~ − 87%) was found in the operated limb and was significantly smaller than those in the contralateral limb after ACLR^20^ and this could remain altered from 1 up to 6 years from ACLR^19^.

Although maximal CSA of individual hamstring muscle appears to be an important index for injury prevention and/or post-surgery monitoring, the calculation of total or partial muscle volume could be regarded as an even more significant measure, as it considers some of the regionality of hypertrophic or atrophic processes^9^. In Fig. 3 it is shown that the individual muscle volume calculated for the portion belonging to the 30–60% of the femur length can be reliably obtained with US when compared to MRI (BFsh showing the least agreement with MRI-based calculations).

### Limitations

The present study has some limitations. When acquiring scans with panoramic US, the results may be influenced by the use of linear transducers on curved surfaces and by the pressure exerted by the operator on the skin and muscle tissue^11^. Although we took care of keeping the transducer always in contact with the skin and keeping the same pressure throughout the whole scan, image distortion may still have occurred. Another limitation is represented by the fact that we assessed only limited muscle portions belonging to the 30–60% of the femur length. However, at such muscle sites the largest CSAs of each individual hamstring muscle are generally located^25,26^. One further limitation could be represented by the choice of focusing on 13–15-year-old athletes. However, we could argue that youth age is clinically wise the most interesting (i.e., onset of overuse injury developments on tendons, first peak in injury rates). Moreover, due to huge difference in biological maturation around the growth spurt, variability among subjects might be bigger than later during the elite stage, thus representing a good sample to investigate the validity and accuracy of US vs. MRI for hamstrings CSA assessment. Nevertheless, as a future direction, further studies should be focused on cohorts of different age and belonging to different sports.

In conclusion, panoramic US is a valid and reliable method for the assessment of individual hamstring muscles CSA at different scan sites along the muscle length. Moreover, it can also provide valid estimations of hamstring muscles volume compared to MRI. A higher agreement (with narrower limits of agreement range) between US and MRI was observed for a trained compared to a novel rater, and, in addition, this agreement was found to improve for the trained rater after the analysis of MRI scans. Our results suggest that panoramic US can be applied in sport and clinical scenarios for the assessment of hamstring muscles size, nonetheless a meticulous training for raters is warranted in order to provide measurements which are truly reliable.
